# Cellular and Molecular Events of Wound Healing and the Potential of Silver Based Nanoformulations as Wound Healing Agents

**DOI:** 10.3390/bioengineering9110712

**Published:** 2022-11-19

**Authors:** Caroline Tyavambiza, Mervin Meyer, Samantha Meyer

**Affiliations:** 1 Department of Biomedical Sciences, Cape Peninsula University of Technology, Bellville 7535, South Africa; 2 DSI/Mintek Nanotechnology Innovation Centre, Department of Biotechnology, University of the Western Cape, Bellville 7530, South Africa

**Keywords:** wound healing, antimicrobial, antioxidant, anti-inflammatory, silver nanoparticles, silver nanocomposites

## Abstract

Chronic wounds are a silent epidemic threatening the lives of many people worldwide. They are associated with social, health care and economic burdens and can lead to death if left untreated. The treatment of chronic wounds is very challenging as it may not be fully effective and may be associated with various adverse effects. New wound healing agents that are potentially more effective are being discovered continuously to combat these chronic wounds. These agents include silver nanoformulations which can contain nanoparticles or nanocomposites. To be effective, the discovered agents need to have good wound healing properties which will enhance their effectiveness in the different stages of wound healing. This review will focus on the process of wound healing and describe the properties of silver nanoformulations that contribute to wound healing.

## 1. Introduction

Wound repair is a complex process that involves the coordinated interaction of various cell types, extracellular matrix molecules (ECM) and soluble mediators such as cytokines, chemokines and growth factors [1]. Wound healing is carefully divided into critical stages that happen simultaneously namely haemostasis, inflammatory, proliferation and remodelling phase [2]. These stages are equally important and failure of any one of them can disrupt the wound healing process and lead to the development of chronic wounds. New wound healing treatments are continually being discovered in a bid to find treatments that are effective in all stages of wound healing. Silver has a long history of use in the treatment of wounds. It has been widely reported to have anti-inflammatory and antimicrobial activities (even against multidrug resistant microorganisms such as methicillin-resistant *S. aureus* (MRSA) and *P. aeruginosa*) [3]. The proposed mechanisms of antimicrobial action of silver and silver nanoparticles (AgNPs) have been described in literature. Briefly, silver ions can interact with sulphur containing proteins, bind to the cell membrane and cause the disruption of the bacterial cell wall. This will eventually affect the permeability of the membrane causing leakage of metabolites, leading to cell death [4,5]. The silver ions also interfere with the electron transport system leading to increased ROS production, protein synthesis inhibition and denaturation of bacterial DNA thus inhibiting cell replication [6,7]. The anti-inflammatory activity of AgNPs are exerted through decreasing the effects of pro-inflammatory cytokines (IL 6, IL 8, IL 1beta, TNF alpha, MMP 9) [8,9,10,11], increasing the expression of anti-inflammatory cytokines (IL 10) [8] and promoting inflammatory cell death via apoptosis instead of necrosis [12]. Many silver-based compounds including colloidal silver (Silver sulfadiazine (SSD)) and nanosilver (AgNPs, nanocrystalline, nanocomposites) have been used to make wound healing creams, bandages and hydrogels [13]. Although these silver-based formulations have improved the healing of wounds, newer formulations that are potentially more effective are continuously being discovered. Since the wound healing process is divided into different stages, it is important to understand the mechanisms of each stage in-order to promote its effectiveness. This review will describe the role of various cell types and molecules in the wound healing process. It will also review how silver containing wound healing agents including AgNPs affect these processes to promote wound healing.

## 2. Cells Involved in Wound Healing

### 2.1. Neutrophils

The wound healing process is orchestrated by different cells and signaling molecules. Neutrophils are the first cells to infiltrate the wound site after injury. The infiltration is facilitated by different signaling molecules and chemoattractants such as damage associated molecular patterns (DAMPs) released by necrotic cells, TGF-β, complement molecules (C3a and C5a), hydrogen peroxide (H_2_O_2_), chemokines and mediators from platelets [14,15]. CXCL8, one of the most common chemokines released by platelets α-granules, together with CXCL1, and CXCL2 play an important role in initiating inflammatory cell recruitment [16]. DAMPs released from damaged cells are known to be first signals to recruit neutrophils after tissue injury. They can directly activate neutrophils by binding to the specific Pattern Recognition Receptors (PRRs) such as toll-like receptors (TLRs) or indirectly by stimulating other cells to release neutrophil chemoattractants [17,18]. At the wound site, the recruited neutrophils also release some pro-inflammatory mediators such as TNF-α, IL-1β, IL-6 and CXCL8 which will recruit more neutrophils and other immune cells thus further promoting inflammation [19]. 

The main function of neutrophils is to prevent infections in the inflammatory phase by clearing the wound of any pathogens, foreign particles, and damaged tissue. They achieve this through phagocytosis, generation of an oxidative burst (due to reactive oxygen species (ROS)) and through the release of destructive proteases, antimicrobial proteins (cathepsins, defensins, lactoferrin, lysozyme) and neutrophil extracellular traps [15,20]. Neutrophils can also promote angiogenesis and the proliferation of fibroblasts and keratinocytes by increasing the expression of the cytokines; VEGF, CXCL3, IL-8, IL-1 β and MCP-1 which promote angiogenesis and proliferation [19]. After completing their task, neutrophils need to be eliminated from the wound site. These cells therefore undergo apoptosis and are subsequently phagocytized by macrophages. The elimination of neutrophils marks the transition from the inflammatory to an anti-inflammatory state [19]. Uncontrolled neutrophil migration prolongs the inflammation process leading to excessive generation of ROS and proteases. The toxic proteases and increased ROS levels degrade the ECM and damages cell membranes leading to prolonged wound healing and formation of chronic wounds [20,21]. 

### 2.2. Macrophages

Macrophages play a fundamental role in all phases of wound healing. It has been proven that the presence of macrophages promotes wound healing. Hu and colleagues (2017) reported that the increase of macrophages accelerated wound healing in both normal and diabetic mice [22]. Macrophages are initially monocytes which differentiate into macrophages after entering the tissues. These monocytes are activated and recruited to the wound site by chemoattractants (such as MCP-1) and DAMP molecules. Pro-inflammatory macrophages also referred to as M1 macrophages are involved in the inflammatory phase of wound healing while the M2 macrophages or anti-inflammatory macrophages are involved in the later stages in wound repair [23]. M1 macrophages infiltrate the wound site, 24–48 h after injury. These macrophages are highly phagocytic, they clear the wound area by phagocytosing bacteria, debris and apoptotic neutrophils (efferocytosis) [24]. M1 macrophages activates other inflammatory cells by releasing pro-inflammatory cytokines (TNF- alpha, IL-6, and IL- beta), and growth factors (PDGF, VEGF and TGF-β1) [25]. They also release matrix metalloproteinases (MMPs) which digests the ECM, making room for infiltrating inflammatory cells and aiding migration [15]. This exacerbates efferocytosis and the pro-inflammatory state of the wound. Successful efferocytosis marks the resolution of inflammation and promotes the switch of macrophages from a pro-inflammatory to an anti-inflammatory state [26]. 

M2 macrophages dominate the anti-inflammatory phase of wound healing. They suppress inflammation by upregulating the expression of pro-resolutory cytokines such as IL-4, IL-10, and IL-13 [27]. They also release growth promoting growth factors including arginase 1, an important factor for effective wound repair and MMPs (MMP-12 and MMP-13) which remodel and strengthen the ECM [24]. Anti-inflammatory macrophages promote new vessel formation, angiogenesis, re-epithelialization, and the transition of fibroblasts to myofibroblasts [15,23]. Recent studies suggest that macrophages are also involved in wound resolution, the final phase of wound healing. This involves the release of anti-angiogenic factors, phagocytosis of apoptotic endothelial cells and the maturation of the epithelium [23,28].

### 2.3. Fibroblasts and Keratinocytes 

Fibroblasts which form the major cellular component of the dermis are the key cells in the proliferation phase of wound healing. They are activated by the release of inflammatory signals mainly PDGF and TGF-beta from platelets and macrophages [14,25]. At the wound site, fibroblasts proliferate and synthesize type 1 and III collagen. They also secrete precursors for components of the ECM including hyaluronan, fibronectin, glycosaminoglycans and proteoglycans. Accumulation of the ECM is essential for the repair process as it supports cell migration [2]. Moreover fibroblasts can differentiate into myofibroblasts causing the wound to contract by contracting the wound bed and bringing the wound edges together [14]. 

Activated fibroblasts secrete paracrine factors such as FGF-2 and KGF which signals adjacent keratinocytes. Keratinocytes respond to these signals by producing PDGF which further stimulates fibroblasts. This kind of interaction between 2 cell types is known as cross talk [29]. Keratinocytes are the predominant cells in the epidermis, their main function in the proliferation phase is re-epithelialization. This is a crucial process which is responsible for restoring an intact epidermis after injury. Epithelialization is a multi-step process involving the proliferation, migration and differentiation of keratinocytes [30,31]. Keratinocytes also stimulate and coordinate the actions of other cell types involved in wound healing. They induce endothelial cell migration and angiogenesis via the secretion of angiogenic growth factors such as VEGF and PDGF [29,32].

## 3. Role of Cytokines and Growth Factors in Wound Healing

Wound healing is a complex process that involves several cell types and is controlled by various molecules, essentially cytokines and growth factors. The release of these cytokines and growth factors is programmed for each of the different phases of wound healing. Any disruption to this carefully orchestrated process may lead to the formation of non-healing chronic wounds. After injury, the pro-inflammatory cytokines, IL-1β, TNF-α and IL-6 are released to attract inflammatory cells to the wound site [21]. Chemokines such as CXCL8 (IL8), CXCL1, and CXCL2 are also released to help this process of chemotaxis [16]. At the wound site, inflammatory cells mainly macrophages release the growth factors, PDFG and TGF-beta which will recruit fibroblasts, initiating the proliferation phase. Active fibroblasts and macrophages release FGF-2 (bFGF), KGF (FGF-7), EGF, HGF, TGF-α and IGF-1 to stimulate keratinocytes which are essential in epithelization [25,29,33]. Fibroblasts, keratinocytes and macrophages further releases VEGF and PDGF to activate endothelial cells and initiate the process of angiogenesis [25]. As wound healing is a continuous and overlapping process most cytokines and growth factors function in more than 1 phase. There are also other signaling molecules involved in wound healing. Table 1 shows a summary of the cytokines and growth factors involved in wound healing.

## 4. Gene Expression in Wound Healing

Wound healing is orchestrated by various genes which code for different signaling molecules (cytokines, chemokines and growth factors) at the different stages of wound healing. The gene expression profile at the site of the wound varies during the different stages of wound healing. The genes are up or down regulated at different stages thus varying the influx and efflux of signaling molecules at the wound site. During the inflammatory phase, the expression of genes which code for pro-inflammatory cytokines, some chemokines and growth factors such as IL-6, TNF-a and IL-8 are upregulated while anti-inflammatory genes such as IL-10 are downregulated [42]. Pro-inflammatory genes coding for TNF activation markers, interferon (IFN) activation markers, leucocytes and macrophage markers were expressed in skin biopsies collected 2 days after wounding in basal cell carcinoma patients [43]. A study by Kubo et al. (2014) showed that the expression levels of IL-1b, IL-6, TNF-a, IFN-c and KC increased 3 h to a day after a skin burn injury in murine models [44]. This is well in the inflammatory phase which is known to take place between 1 h and 3 days after injury [45]. Moniri et al. (2018) reported that treating human dermal fibroblast cells with bacterial nanocellulose/silver (BNC/Ag) nanocomposites increased the expression of TGF-β1 from 4.8- to 11-fold at 6 and 24 h, respectively [46]. TGF-β1 is involved in almost all the processes of wound healing. It prompts the recruitment of inflammatory cells into the injury site, improves the angiogenic properties of endothelial cells, stimulates fibroblast contraction and promotes keratinocyte migration [47,48]. The presence of TGF-β1 is certainly of great importance in the wound healing process. In fact, it was stated that the chronic, nonhealing wounds often show a loss of TGF-β1 signaling [49]. However, prolonged release of this molecule can lead to hypertrophic scar formation. Hypertrophic derived fibroblasts were shown to have prolonged expression of TGF-β1 and TGF-β receptors compared to normal skin fibroblasts. In normal wound healing, the expression of TGF-β receptors decreases during the remodeling phase [49,50,51]. 

As wound healing proceeds, the expression of genes changes from a pro-inflammatory to that of a repair profile. This gene profile includes genes that promote fibroblasts and keratinocytes proliferation as well as granulation and epithelialization, these include VEGF, PDGF and FGF-2 [42]. Deonarine et al. (2007) showed that even though the proinflammatory genes were increased early (2 days) in the wound, after 4 to 8 days the profile of expressed genes changed to those of repair and angiogenesis. In this study, after day 4 and 8, the expression of type IV collagen, procollagen, integrin αv, integrin β5, MMP-2, MMP-9 and progranulin was increased [43]. These genes are involved in proliferation and maturation which occurs between approximately 4 to 21 days of wound healing. Kubo et al. (2014) reported similar results, in which the expression of VEGF, MMP-2, MMP-13 and type I collagen increased after 3 to 14 days in a skin burn injury [45]. Unlike pro-inflammatory cytokines released 3 h after wounding, IL 10 was released after 12 h and lasted up to 7 days post wounding [44]. The remodelling phase, which is the last phase of wound healing, is responsible for the development of new epithelium through restoration of tissue architecture and tissue strength [14]. In this phase, most wound healing genes are downregulated however TGF-β1 and MMPs are upregulated. TGF-β1 stimulates fibroblasts to produce type I and III collagen, while MMPs are responsible for the degradation of collagen. The activity of metalloproteinases is however tightly regulated as it might degrade essential collagen thus causing impaired healing [2,42]. Any disturbances (overexpression, under expression or prolonged expression) in the expression of genes and release of cytokines, chemokines and growth factors in wound healing will disrupt the sequence of healing which may lead to the development of chronic wounds. 

## 5. Uses of Silver in Wound Healing 

The effectiveness and the historic use of silver to control microbial infections have led to its incorporation in chronic wound treatment. Many silver containing products such as wound dressings have been developed for topical wound treatment [4]. Earliest silver preparations used for wound treatment were SSD and silver nitrate. SSD was the standard treatment for burn wounds. However, these preparations provide very high levels of the silver ion (3176 ppm), much higher than the therapeutic range (30–60 ppm) [52,53]. Due to increased silver ion levels, SSD have been reported to cause significant cellular cytotoxicity [52]. Nano formulations of silver contain lower concentrations of the silver ions and are active at lower concentrations (approximately 70 ppm), making them more suitable for wound treatment [53]. They have been shown in many instances to be more effective than SSD [54,55,56]. In addition to the antimicrobial activity, silver nanoformulations were reported to enhance anti-inflammatory responses, stimulate proliferation and migration of keratinocytes and fibroblasts and to improve collagen expression and formation [11,57,58,59]. Combining silver and nano-silver formulations with other wound treatments has been shown to have synergistic effects. Silver ions were reported to increase the activity of negative wound pressure therapy with polyurethane sponge by reducing biofilm, increasing bacterial inhibition, and reducing healing time and the cost of treatment [60,61,62,63]. Nanocrystalline wound dressing were reported to be more effective than the standard dressings. They ensure a sustained release of constant silver ions at therapeutic dose, which reduces the need to change dressings. This reduces disruption to the wound healing bed and minimizes patient discomfort and pain [7,53]. Unfortunately, silver nanoformulations do show some toxicity although to a smaller extent. Therefore, thorough toxicity studies should be done for all nanoformulations and they should be used for a limited time and only when necessary.

## 6. Properties of a Good Wound Healing Agent

The properties of good wound healing agents would include antimicrobial, anti-inflammatory, angiogenesis, antioxidant activity, as well as the ability to promote procollagen synthesis. They also promote the growth, differentiation and migration of fibroblasts and keratinocytes.

### 6.1. Antimicrobial Activity

It is essential for any wound healing agent to have antimicrobial activity. Infections particularly bacterial infections are one of the main causes of delayed wound healing. Bacteria have been more frequently isolated form wounds compared to other microorganisms such as fungi. Injury breaks the skin barrier that protects the body against microbial colonization, resulting in the exposure of the underlying tissue to various infectious organisms [64]. The skin impairment gives bacteria access to the wound site where they infiltrate, contaminate and colonize the wound. Contamination is defined as the presence of non-replicating bacteria. Contamination can lead to colonization which involves the active replication of microorganisms without eliciting an immune response [65]. Generally, all wounds are contaminated with bacteria because they provide a favorable medium for bacterial growth [66]. However, the bacterial load in the wound changes as contamination progress to colonization and finally infection. In most cases a bacterial load of more than 10^5^ viable bacteria per g of wound tissue is considered as an indication of infection [67,68]. Infection occurs when the microorganisms continue to replicate to the extent of invading and damaging soft tissues thus triggering an immune response [69]. 

Gram positive, coagulase-negative Staphylococci (e.g., *Staphylococcus aureus*) which are usually normal skin flora are the first to enter the wound site after disruption of the intact skin barrier. This is followed by the invasion with Gram negative aerobes such as *Escherichia coli* and *Pseudomonas aeruginosa*. These aerobes reduce the oxygen levels in the wound environment promoting the invasion of anaerobes [70]. Therefore, as the wound matures into a chronic wound, anaerobes are frequently present at the wound site. Most chronic wounds contain mixed populations of microorganisms, the most common bacterial strains found at the wound site include *S. aureus*, MRSA, beta-haemolytic Streptococci, *E. coli*, *P. aeruginosa* [69,71,72]. 

Bacteria release toxins and proteases which can damage surrounding tissue cells in the wound area [66,73]. These toxins can also elevate the levels of pro-inflammatory cytokines thus prolonging the inflammatory phase. Due to sustained inflammation, the balance between proteases and protease inhibitors is disrupted resulting in increased levels of proteases. These proteases degrade the ECM and growth factors, hindering cell migration and prevent wound closure [65,71,74]. Pathogens can stick together and form biofilms which can be described as aggregations of microbial cells embedded in a polymeric matrix called extracellular polymeric substance (EPS). Due to the EPS layer, biofilms can evade the activity of antibiotics and host defenses thus protecting the embedded pathogens [64]. 

Antimicrobial activity is one of the properties for an effective wound healing agent. All wound healing agents have been shown to possess antimicrobial activities. Silver is known for its good antimicrobial activity, it has been used in the medical field since the 1900s. Due to its antimicrobial properties, its incorporation in wound healing was inevitable. Different silver-based formulations have been used in wound healing; these include SSD and newer formulations such as Aquacel, Silverlon and Acticoat [52]. Many AgNPs, although at a research level, have proved to be promising wound healing agents [75]. Various studies have proved the antimicrobial properties of these compounds. 

SSD which is used in the treatment of burn wounds is a broad-spectrum antimicrobial agent active against *S. aureus*, *P. aeruginosa* and *E. coli* [4,54]. However, it does have some shortcomings, which is why wound treatments containing nanocrystalline are being developed. Nanoformulations of silver are more effective because of their smaller size which increases surface area thus increasing activity. Silver-coated polyurethane sponges were shown to reduce bacterial counts of biofilm-causing organisms such as *S. aureus*, *P. aeruginosa* and MRSA [52]. Electrospun nanofibres (MADO) integrated with AgNPs were shown to be effective against the bacteria such as *S. aureus*, *P. aeruginosa* and *E. coli* [76]. Nanocomposites containing AgNPs showed significant antimicrobial activity against *S. aureus* and *E. coli* [77,78]. Many biogenic AgNPs have also been reported to have antimicrobial activity against various microorganisms [11,79,80,81,82].

### 6.2. Anti-Inflammatory Activity

Inflammation is the immune system’s defense mechanism against various threats to the body. It is a complex process demanding the participation of both the vascular and cellular elements of the immune system. Inflammation can be either acute or chronic. Acute inflammation is short-lived, lasting for hours to days only and normally resolving after the cause of injury has been eliminated [83]. It is characterized by key features at the injury site commonly known as the cardinal signs of inflammation. These features include redness (rubor), swelling (tumor), heat (calor), pain (dolor) and loss of function (functio laesa) [84,85]. The redness and heat result from vasodilatation and increased blood flow to the inflamed site, the swelling is caused by increased vessel permeability causing leakage of fluid and plasma proteins into the interstitial area and the pain is due to an intensive sensation of harmful stimulus [84,86,87].

Any harmful stimuli to the body can incite an inflammatory response. Infection by microorganisms such as bacteria, viruses and fungi can cause inflammation [88]. Non-infectious causes of inflammation can be categorized as physical (burns, frostbites, physical injury, foreign bodies, trauma), chemical (hydrolytic enzymes, glucose, acids, toxins,) and biological [84,88]. The immune system recognizes noxious stimuli, both infectious and non-infectious, using PRR [86]. During microbial infection, PRRs identify the invading microorganisms using structures known as pathogen-associated molecular patterns (PAMPs). In non-infectious conditions, PRRs can recognize endogenous molecules released from damage cells named DAMPs [89,90]. There are different classes of PRRs including the TLRs, C-type lectin receptors (CLRs), Retinoic acid-inducible gene (RIG)-I-like receptors (RLRs) and NOD-like receptors (NLRs). Amongst all these, TLRs are the most extensively studied PRRs, they can bind to either PAMPs or DAMPs to initiate an inflammatory response [86,91,92].

Acute inflammation is an essential step in wound healing. It is characterized by brief vasoconstriction as an attempt to reduce blood loss after injury. Followed by vasodilation which increases the permeability of blood vessels causing leakage of plasma proteins and allowing influx of inflammatory mediators (including chemokines, cytokines, free radicals). These mediators promote leucocytes migration from the blood vessels to the site of inflammation (wound site) [93]. Neutrophils are the first cells to infiltrate the wound site followed by monocytes and lymphocytes. At the wound site monocytes differentiate into macrophages and secrete chemokines and pro-inflammatory cytokines such as IL-1, IL-6 and TNF-alpha which further promoting the inflammatory process [94]. The recruited immune cells clear off the antigens through phagocytosis and the release of ROS. Once the noxious stimulus is removed and the damage is repaired, the inflammatory response should be resolved to prevent transformation into chronic inflammation [86,93]. Mononuclear cells (macrophages, lymphocytes, and plasma cells) especially macrophages replace the pre-existing neutrophils and become the prominent cells in chronic inflammation. This type of inflammation is associated with prolonged tissue damaged, tissue granulation and fibrosis [95]. Prolonged or chronic inflammation can impair wound healing resulting in chronic wounds such as diabetic wounds [96], it can also lead to the development of various diseases such as atherosclerosis, cardiovascular diseases, and cancer [97]. 

Although inflammation is an essential stage of wound healing, prolonged inflammation can impair the wound healing process and lead to the development of chronic wounds [98]. Anti-inflammatory treatments can be of importance when added to wounds; they can inhibit prolonged inflammation and assist the wound to change from its inflammatory phase to the healing phase [45]. Chronic inflammation delays the proliferation and migration of fibroblasts and keratinocytes thus delaying the process of proliferation and re-epithelization [58]. Chronic wounds are characterized by elevated levels of pro-inflammatory cytokines. Diabetic wounds become stuck in the inflammatory phase, thus disrupting the formation of growth factors and cytokines (e.g., IL10) needed for the proliferation and maturation phases [99]. Silver and AgNP based wound treatments as well as some biogenic AgNPs have been reported to possess anti-inflammatory activity. Nanocomposites made from bamboo cellulose matrix infused with AgNPs were reported to exert significant anti-inflammatory activity in mice [57,58]. The nanocomposites notability decreased the levels of the pro-inflammatory cytokines, TNFα and IL-6 and in turn shortened the wound healing time compared to the controls [57,58]. 

Nanocrystalline silver coated dressings were also reported to have good anti-inflammatory properties. In a study by Tian et al. (2007), AgNP grafted wound dressings reduced pro-inflammatory cytokine IL-6 levels while increasing levels of the anti-inflammatory cytokine IL-10 in 20 week old mice (BALB/C) [8]. In a porcine model of contact dermatitis, nanocrystalline silver dressings decreased inflammation by decreasing the levels of pro-inflammatory cytokines (TNFα, IL-8, MMP9), decreasing gelatinase activity and increasing apoptosis of inflammatory cells [10]. Death of inflammatory cells by necrosis causes the cells to burst and release inflammatory contents such as proteases and oxygen radicals which further increases inflammation. On the other hand, cellular death by apoptosis allows the cells to maintain the plasma membrane and inhibit the release of inflammatory contents [100]. Apoptosis of inflammatory cells therefore plays a role in resolving local inflammation in dermal wounds [12]. In another porcine study by Wright et al. (2002), nanocrystalline silver coated dressings were also effective in reducing the levels of MMPs and reducing the number of infiltrating mononuclear cells via apoptosis [12]. Controlling inflammation can therefore allow rapid wound healing as well as faster epithelization and maturation of the repaired tissue.

### 6.3. Antioxidant Activity

Antioxidants are known to promote the wound healing process by reducing the effects of oxidative stress in the wound [101]. They protect biological molecules such as DNA, proteins and lipids from damage by excessive amounts of ROS [102]. Although low levels of ROS and oxidative stress are important in the normal physiology of wound healing, the presence of excessive amounts can be damaging and can lead to wound healing impairment. It is therefore imperative to have a balance between the pro-oxidative and anti-oxidative systems in the body [103].

Reactive oxygen species are by-products of the normal oxygen metabolism in the body [104]. They include free radicals such as superoxide anion, and hydroxyl radical, and some non-radical molecules such as hydrogen peroxide. ROS are highly reactive and have higher oxidative potential than the molecular oxygen which is relatively stable [105]. The production of ROS is physiological, actually it is estimated that 2–5% of the total oxygen used by humans daily (around 250 g) is converted to ROS [106]. In basal levels, ROS play a significant role in wound healing as mediators of cell signaling and growth regulation [103]. Low levels of ROS are essential in all phases of wound healing namely haemostasis, inflammation, proliferation, and regeneration. During haemostasis, ROS enhances the clotting process by increasing platelet recruitment and aggregation as well as collagen induced platelet activation which promotes clot formation [106,107]. ROS also potentiates the inflammatory process by directly effecting neutrophil chemotaxis to the wound site and supporting the survival of monocytes and macrophages through the expression of monocyte colony stimulating factor-1 (CSF-1) [108]. It is also a defense mechanism that clears the wound site of any invading microbial pathogens. ROS further promotes the inflammatory response by stimulating the release of TNF-α and PDGF [107]. In wound re-epithelialization, ROS promotes fibroblast and keratinocyte proliferation and migration through mediating the signaling of FGF, EGF as well as collagenase (MMP-1) expression. ROS also stimulates angiogenesis and matrix deposition via the expression of FGF-2 and VEGF. In addition, these oxygen species mediate conversion of fibroblasts to myofibroblasts thus aiding wound contraction [106,108,109]. 

ROS levels are strictly controlled and kept at the basal levels by antioxidants including enzymes such as superoxide dismutase (SOD), catalase, glutathione peroxidases and peroxiredoxins and nonenzymatic compounds such as vitamin E and glutathione [107]. Any imbalance between the production of ROS and their destruction by antioxidants leads to oxidative stress. During oxidative stress there is overexposure to ROS which have deleterious effects on wound healing [110]. Increased levels of ROS and free radicals cause severe tissue damage through lipid peroxidation, protein modification and DNA damage. These oxygen radicals break peptide bonds in the backbone of proteins thus changing the protein structure and functionality. They cause lipid peroxidation of membranes at both the cellular and the organelle level by oxidizing important protein and enzyme systems [101,103,111,112]. High levels of ROS affect wound healing in different ways; it impairs angiogenesis, inhibits the proliferation and migration of fibroblasts and prolongs inflammation [106,109]. Prolonged inflammation due to increased stimulation of neutrophil and macrophage chemotaxis and migration further increase the levels of ROS consequently increasing cellular and tissue damage. The levels of protease inhibitors are decreased thereby disrupting the protease-antiprotease balance. The excess protease uncontrollably degrades the ECM components such as collagen and proteoglycans thus delaying wound healing [106]. 

Nanoformulations especially those made from plants with a known history of good antioxidant activity have been shown to exhibit higher antioxidant properties which increases the wound healing activities. AgNPs synthesized using extracts of *Pongamia pinnata* showed better antioxidant potential compared to the plant extract and ascorbic acid [113]. In turn, the nanoparticles exhibited better anti-bactericidal activity and decreased wound healing time more than the extract [113]. Silver nano-colloids synthesized from antioxidant rich aqueous extracts of *Aerva javanica* had good antioxidant potential, they also showed low toxicity in both in vitro and in vivo systems [114]. After their incorporation into hydrogels, the nanoparticles incorporated hydrogels promoted rapid wound healing compared to the negative control [114]. In a study by Hajji and colleagues (2019), chitosan-PVA-silver (CS-AgNPs) nanoparticles exhibited higher antioxidant activity than the chitosan powder. Moreover, the CS-AgNPs were characterized by a low cytotoxicity effect against Chinese Hamster Ovary cells, they were also found to significantly promote wound healing [115]. 

### 6.4. Epithelization

Epithelization is an essential process in wound healing. It is defined as the formation of epithelium over a denuded surface [116]. Without re-epithelization, a wound cannot be considered healed. Impairment of the re-epithelization process will ultimately result in the development of chronic wounds. It also increases the risk of the wounds to infection and loss of fluids, which disrupts the healing process [117]. Epithelization involves the migration of epithelial cells mainly keratinocytes from one wound edge to another, forming a new tissue barrier between the wound and the outside environment [118]. To allow for migration, keratinocytes at the basal layer undergoes changes which cause them to detach from the basement membrane and neighboring cells. They also differentiate from a proliferated state to a non-proliferative state as they migrate through the granular layer [116]. Following keratinocytes migration over the wound bed, the basal keratinocytes behind the migratory cells begin to proliferate to ensure that an adequate supply of cells cover the wound [119]. Successful keratinocyte migration occurs through the fibrin and new ECM synthesized through the aid of fibroblasts. Therefore, effective epithelization occurs in the presence of fibroblasts and some endothelial cells.

During proliferation, fibroblasts accumulate at the wound site and deposit ECM components including collagen type I and III. These are used to produce a new ECM in order to replace the clot that is initially present at the wound bed [119]. ECM provides a platform for keratinocyte migration during epithelization. Some of its components particularly collagen directly enhances keratinocyte migration. The new ECM does not resemble the ECM before injury, it is much looser thus allowing for cellular invasion [42]. Keratinocytes bind to collagen through the α1β2 integrins [117]. As there is formation of new tissue during this repair process, endothelial cells are also recruited to the wound site. They start forming new blood vessels from pre-existing ones, a process called angiogenesis [120]. Angiogenesis provides the oxygen and nutrients needed for growth of new tissue during repair. For effective wound repair, these core wound healing processes (fibroplasia, epithelization, and angiogenesis) need to occur successfully. 

A good wound healing treatment should facilitate the processes of fibroplasia, angiogenesis, and epithelization. Various silver and nanosilver-based wound healing agents have been shown to enhance keratinocyte migration, fibroblast proliferation, collagen deposition and angiogenesis. Some of the functions in the proliferation and maturation phases of wound healing are included in Table 2. 

### 6.5. Biocompatibility

Toxicity is one of the most important factors determining the effectiveness of a wound healing agent. For effective healing, the wound healing agents being investigated should not be toxic to the normal cells surrounding the wound. The treatments need to have selective toxicity, being toxic only to microorganisms and damaged cells and not to normal healthy cells. It has been reported that damage to keratinocytes and fibroblasts impairs wound healing. Most common wound therapies such as negative pressure wound therapy, hyperbaric oxygen and administration of growth factors can be toxic to tissues and are therefore associated with complications such as bleeding and pneumothorax [13,75]. They are also very costly therefore silver-based therapies which are more cost-effective were investigated as alternative therapies. Silver-based therapies have promising wound healing properties, they have a broad antimicrobial spectrum, are good anti-inflammatory agents and were shown to shorten the wound healing time [13]. However, despite their popularity, some silver based wound therapies were also shown to be toxic to normal cells. Toxicity of silver-based products has been attributed to the release of the silver ions. Silver diffuses through the wound into the skin cells and causes a grey skin discoloration known as argyria [13,122,123,124]. In some cases, the prolonged use of silver products may also cause epithelial and fibroblast toxicity especially if high concentrations of silver are used [7,125,126,127]. Systemic toxicity is however rare because silver ions can be neutralized by anions in body fluids [75]. The more silver ions are released, the higher the toxic effects they present. For example, SSD, the widely used treatment for burn wounds has been found to release high levels of silver (3.176 ppm) into the wounds [52]. It has been reported that SSD has high local toxicity and can also cause bone marrow toxicity because of the presence of propylene glycol. Newer silver formulations in the form of nanoparticles and nanocrystalline are being used for wound healing. These formulations were found to be more effective while being less toxic as they use much lower concentrations of silver. AgNPs and nanocrystalline silver were shown to be more effective and less toxic than SSD and silver nitrate in various studies [52,54,55,56]. The toxicity of AgNPs has however been reported, AgNPs exert their toxicity through ROS generation, protein oxidation and membrane damage [128]. Nanoparticle toxicity is determined by their characteristics with the most important one being the size [129]. In most cases smaller nanoparticles are more toxic than the larger ones, smaller nanoparticles can easily enter into cells and pass-through biological membranes thus having a higher chance of causing cellular toxicity [128,130]. It is therefore important to thoroughly investigate the toxic effects of these nanoformulations before using them for wound treatments. The toxicity of Ag and AgNPs has been extensively covered in other review articles [52,123,128,129,130,131,132,133]. 

## 7. Incorporation of the Silver Formulations in Wound Dressings

The incorporation of wound healing agents into wound dressing can greatly facilitate wound healing. An ideal wound dressing is one that protects the wound from external contamination, is biocompatible and semipermeable to oxygen and water [134]. All this keeps the wound moist, which facilitates epithelialization in wound healing. An ideal wound dressing should also be cost effective, non-toxic and hypoallergenic, it should be nonadherent and can be easily removed without trauma [4]. Traditionally used wound dressings such as the gauze, cotton and wool cannot sufficiently provide wound care requirements, they can cause drying out of wounds and lead to trauma upon removal [4,58]. Therefore, newer dressings made from biodegradable materials such as chitosan, hyaluronic acid, collagen, silicon, cellulose, and gelatin are being used. These new materials do not only preserve the wound environment, they also release bioactive compounds in the wound and aid in the process of healing [124,134]. Incorporation of silver and its nanoformulations in wound dressings have been sown to enhance wound healing. Silver and nano-silver wound dressings have been reported to have; significant antimicrobial activity, decrease inflammation, improved collagen expression and increased epithelial cell differentiation, proliferation and migration [8,57,58,76,77,78]. All these properties allow the silver wound dressings to improve the wound healing process leading to faster wound healing compared to normal dressings. Silver wound dressings have also been associated with reduced pain and anxiety as well as lower treatment costs thus improving patients’ lifestyle [55,135]. Many other silver and nano-silver formulations are still to be explored. Good examples are the biogenic AgNPs synthesized using medicinal plants known to possess wound healing activities. A few of these, including AgNPs synthesized from *Cotyledon orbiculata* [11], and *Garcinia indica* [136] have been reported in literature.

## 8. Conclusions

Wound healing is a complex process that is carefully divided into critical stages which happen simultaneously. It is a delicate process in which failure of any one of them can disrupt the whole wound healing process and lead to the development of chronic wounds. Wound healing agents need to have good wound healing properties which will enhance their effectiveness in the different stages of wound repair. Good wound healing agents have properties such as antimicrobial, anti-inflammatory, angiogenic and antioxidant activities. They can also promote the growth, differentiation and migration of fibroblasts and keratinocytes. Different silver nanoformulations were shown to enhance wound healing at different stages. Most of the formulations were effective in more than one stage of healing however none of them was effective in all the wound healing stages. Combination therapy in which one or more compounds are combined in the treatment of a wound, or where different compounds are applied to the wound based on the stage of wound healing may be our answer to effectively treat chronic wounds. It is therefore of great importance to continuously research and develop different therapies and combination therapies that can successfully combat problematic chronic wounds. 

## Figures and Tables

**Table 1:** Cytokines and growth factors involved in wound healing and their functions.

Growth Factor/Cytokine	Function	Reference
IL-1β, TNF-α and IL-6	Inflammation	[34]
PDGF	Chemotaxis of neutrophils and macrophages. Proliferation of fibroblasts. Induces myofibroblasts differentiation. Upregulates the production of insulin growth factor 1 (IGF-1). Stimulate angiogenesis.	[35,36]
TGF-beta	Chemotaxis of neutrophils and macrophages. Fibroblast proliferation. Myofibroblast differentiation. Stimulate re-epithelialization. Stimulate angiogenesis.	[25,37]
TGF-alpha	Stimulates proliferation and migration of keratinocytes. Induces angiogenesis.	[38,39]
bFGF (FGF-2)	Increases keratinocyte motility. Promotes the migration of fibroblasts and aids in tissue remodeling.	[2,40,41]
KGF	Stimulate keratinocyte differentiation and proliferation.	[25,39]
EGF	Promotes fibroblast and keratinocyte growth. Stimulate the proliferation and migration of keratinocytes.	[38,39]
IGF	Increases keratinocyte motility and promotes fibroblast growth.	[35]
VEGF	Increases endothelial cell migration and proliferation. Promotes angiogenesis.	[32,40]

**Table 2:** Silver based wound healing agents reported to enhance the proliferation phase of wound healing.

Wound Healing Agent	Function in Proliferation and Maturation Phase	References
Hydrogels (prepared from bamboo cellulose nanocrystals impregnated with AgNP)	Improved epithelialization. Improved collagen formation. Increased expression of collagen and growth factors (FGF, PDGF, VEGF). Improved vasculogenesis.	[57,58]
*Pongamia pinnata* seed extract-AgNPs loaded gel	Shortened wound healing time compared to other groups. Showed injury recuperating action, which might be because of their angiogenic and mitogenic potential	[113]
Muslin cloth coated with *Delonix elata*-AgNPs	Showed rapid wound epithelialization compared with the control.	[121]
Partially carboxymethylated cotton gauze (PCG) with AgNPs	Promotes fibroblast generation. Promotes neovascularization. Promotes formation of granulation tissue. Enhances epithelialization.	[77]
AgNP-solution-coated dressing	Increased keratinocyte proliferation and migration. Facilitates fibroblasts differentiation. Shortened wound healing time compared to other groups.	[59]
Electrospun nanofibres (MADO) integrated with AgNPs	The wound treated with MADO-AgNPs showed a complete glandular cavity, thickened epidermis, granular tissue formation, and keratinocyte restoration.	[76]

## Data Availability

Not applicable.

## Abbreviations

AgNP	silver nanoparticle
CLR	C-type lectin receptor
CSF-1	colony stimulating factor-1
DAMP	damage associated molecular pattern
ECM	extracellular matrix molecules
EGF	epidermal growth factor
FGF	fibroblast growth factor
IFN	interferon
IGF	insulin growth factor 1
IL	interleukin
KGF	Keratinocyte growth factor
MMP	matrix metalloproteinase
NLR	NOD-like receptor
PAMP	pathogen-associated molecular pattern
PDGF	platelet-derived growth factor
PRR	pattern recognition receptor
RLR	Retinoic acid-inducible gene (RIG)-I-like receptor
ROS	reactive oxygen species
SOD	superoxide dismutase
SSD	silver sulfadiazine
TGF	Transforming growth factor
TLR	toll-like receptor
TNF-α	tumor necrosis factor alpha
VEGF	vascular endothelial growth factor
